# Impact of Physicians’ Competence and Warmth on Chronic Patients’ Intention to Use Online Health Communities

**DOI:** 10.3390/healthcare9080957

**Published:** 2021-07-29

**Authors:** Xijing Zhang, Runtong Zhang

**Affiliations:** Department of Information Management, School of Economics and Management, Beijing Jiaotong University, Beijing 100044, China; 20113055@bjtu.edu.cn

**Keywords:** online health community, chronic disease, competence, warmth, the technology acceptance model

## Abstract

In China, medical resources are unevenly distributed, and hospitals are very congested. Online health communities (OHCs) provide a new way for patients to communicate and obtain health-related information, thereby alleviating the pressure of treatment in hospitals. However, little is known about how to increase individuals’ use intention for OHCs from the perspective of physicians. This study aims to investigate the impact of physicians’ competence and warmth on chronic patients’ intention to use physician-centered OHCs based on the technology acceptance model. A formal investigation was anonymously conducted through a web-based questionnaire survey addressed to participants, and 710 valid responses were received. A research model was constructed and the hypotheses were tested by structural equation modeling. The findings suggest that competence and warmth positively affect chronic patients’ behavioral intention to use (BIU) OHCs through the mediation of perceived usefulness (PU) and perceived ease of use (PEOU). All hypotheses were supported at the 0.05 significant level. Compared with competence, warmth has a slightly stronger impact on PU and PEOU. PEOU has a stronger impact on chronic patients’ BIU OHCs than PU. This study provides a comprehensive understanding of the impacts of physicians’ characteristics in physician-driven OHCs. Compared with competence, physicians’ warmth should be paid more attention to motivate more chronic patients to use OHCs. Enhancing physicians’ warmth and the ease of use are the preferred ways to improve chronic patients’ intention to use OHCs.

## 1. Introduction

The shortage and unequal distribution of medical resources in China lead to a lack of effective and continuous communication between physicians and patients. Online health communities (OHCs) are virtual social networks where individuals can share health experiences, post health questions, seek, and/or provide emotional support [1]. They contribute to improving the quality and efficiency of medical resources and hold promise in the future for the health industry. OHCs are also an important means to manage chronic diseases. Chronic diseases are known as “silent epidemics” and affect an increasing number of people every year, causing serious financial burden. Chronic diseases have become a serious public health and social problem that endangers the people’s health and the sustainable development of society and the economy [2]. The continuous spread of chronic diseases has resulted in a heavy economic burden on the country. Therefore, the prevention and control of chronic diseases and treatment are significant. Researchers state that eHealth can reduce healthcare costs and improve healthcare outcomes [3]. A Pew survey showed that 61% of American adults search online for health information [4], and patients with chronic disease have become more dependent on OHCs [5]. The reasons are as follows: (1) Chronic diseases take a long time to cure, and thus, patients need continued interactions with physicians, with OHCs presenting a convenient and effective way for them to consult physicians on the Internet. (2) Chronic diseases always need to be improved by changing the person’s lifestyle and eating habits, changes that must be maintained, indicating the need for a comprehensive understanding of their health condition. Contrary to this, most physicians in hospitals instruct patients how to follow treatment plans without fully explaining the reasons in detail [6]. (3) Chronic patients can learn more about their health problems through social support from OHCs [7]. Further, a physician-driven OHC may be better suited to the self-management of chronic diseases than traditional patient-oriented OHCs because it enables patient–physician relationships and generates better self-management support [8]. With growing numbers of chronic patients turning to the Internet for health information and social support, patients’ decision-making processes and their behavioral intention to use (BIU) OHCs must be understood [9]. The use intention can translate into effective utilization of OHCs, thereby combating the growing burden of chronic diseases.

Compared with patient-centered OHCs, physician-centered OHCs have gradually received attention as they can be a better choice than traditional patient-oriented OHCs for chronic diseases [8]. Physician-centered OHCs enable patients to form partnerships with physicians and generate better self-management support by integrating the physician’s medical expertise with the patient’s experience [8]. In physician-centered OHCs, each physician has their own homepage of an online medical community. They can provide medical services online through the platform. Patients can also schedule an appointment to consult with the physicians and view articles provided by the physicians beforehand. Given their role in establishing OHCs, physicians play a role in guaranteeing the credibility of the medical information provided, thus reducing the redundancy and improving the quality of medical information in the online medical community [10]. In physician-centered OHCs, physicians’ characteristics are critical for patients’ decisions on whether to use these online platforms [1]. The physicians’ quality and traits have a positive effect on OHCs’ quality and patients’ intention to use these platforms [11].

People do not simply judge others from a “bad to good” dimension. Psychological research involving thousands of people from different cultural backgrounds has determined that two characteristic dimensions—namely, competence and warmth [12,13]—can be used to “categorize” our social world. Competence and warmth are traits that dominate the social judgments of individuals and groups, thus shaping emotions and behaviors. Stereotypes of individuals are captured by competence and warmth [13,14]. Classifying people based on competence and warmth is a universal dimension used to explain interpersonal and intergroup social cognitions. Thus, people perceived as competent and warm elicit uniformly positive emotions and behavior [13]. Consumers’ judgments of competence or warmth influence their purchase intention, loyalty and brand recommendation behavior [15]. However, no study has investigated the effect of physicians’ competence and warmth on chronic patients’ attitude toward OHC use. Thus, this study attempted to investigate physicians’ effect on chronic patients’ intention to use OHCs from the perspective of competence and warmth. The technology acceptance model (TAM) is generally used for determining individuals’ intention to use technology [16]. The TAM, especially, has become an important theoretical model for studying medical information usage behavior [17]. TAM theory has been leveraged for more general measures of the factors affecting patients’ BIU to use OHCs.

This paper constructs a comprehensive and encompassing model of how physicians’ traits, i.e., competence and warmth, influence chronic patients’ BIU OHCs based on TAM theory, thus offering an in-depth understanding of the complex behavior of chronic patients. Specifically, the independent variables are physicians’ competence and warmth, the mediating variables are perceived usefulness (PU) and perceived ease of use (PEOU), and the dependent variable is chronic patients’ intention to use OHCs. Through a web-based questionnaire survey, 710 responses, which came from those who had received treatment within the previous three months in OHCs, were collected. Structural equation modeling and SmartPLS version 3.2.8 were used to test the relationships. 

## 2. Research Model and Hypotheses

### 2.1. TAM

The TAM was introduced by Davis; it shows how technology users will accept and use a new technology [18]. The TAM can predict the usage, individual intention to perform specific actions, and individual user acceptance of information systems and technologies [18,19]. TAM theory contains two constructs to understand information technology (IT) behavior: PEOU and PU. The PEOU represents the degree to which a person believes that a particular system is easy to use; the PU [18] represents “the degree to which a person believes that using a particular system would enhance his or her job performance.”

TAM theory has been demonstrated in multiple research fields, such as online education systems [20], social networking sites [21], and e-banking systems [22]. In particular, the TAM is one of the most popular research models in telehealth areas [23]. OHCs are Internet-based platforms for users to exchange health information and seek support from physicians. They are a type of virtual community, developed with Web 2.0 technology. Thus, the TAM can be used in online health care field. This study used TAM theory to analyze the effect of physicians’ competence and warmth on patients’ intentions, and provides business insights into how OHCs can attract more consumers by increasing the numbers of convincing physicians leading the communities.

### 2.2. Research Model

**Figure 1:**
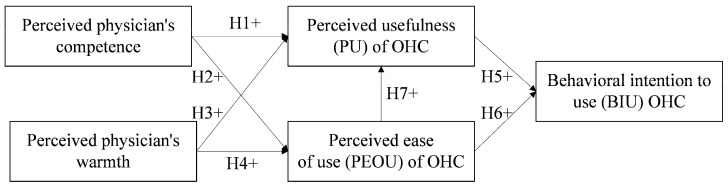
Research model. H1+, H2+, H3+, H4+, H5+, H6+, H7+ represent positive hypotheses.

As shown in Figure 1, the independent variables are physicians’ competence and warmth, the mediating variables are PU and PEOU, and the dependent variable is chronic patients’ BIU OHCs. 

### 2.3. Competence

McClane, for the first time, came up with a definition of competence as a potential personal trait that separates the high performers from the low performers in a given job [24]. Competence is used to evaluate the degree and possibility of others achieving their intentions and goals, and it is represented as intelligence, skill, efficiency, creativity, confidence, and cleverness [13]. Thus, competence is an important factor that reflects therapists’ abilities to make treatment plans and set the outcomes. In the health industry, health services are associated with mortality and fundamental quality of life [25]. Physicians’ skills are more important to patients than other kinds of service [26]. Thus, in the context of OHCs, we relate competence to the professional competence in the role of being a physician. We assume that physicians’ competence can positively affect chronic patients’ perception of usefulness and ease of use. 

First, as the main provider of medical services, physicians’ competence [27] determines whether the medical service provided is qualified, whether the patient is satisfied, whether the diagnosis and treatment result are useful, and whether the work efficiency is high. Therefore, physicians’ competence largely determines the quality and level of the medical service supplied. Physicians with high competence have sufficient professional knowledge and abilities to provide health-related services to cure patients. Meanwhile, physicians’ professional abilities impact health information quality and further impact the treatment outcome [8]. In other words, physicians’ competence can guarantee the health information quality in physician-centered OHCs. If physicians in OHCs can make medical decisions independently and cure patients’ diseases, then patients will find physician-center OHCs useful for improving their health condition [28]. Thus, competence and professional service are associated with service quality, which can influence users’ PU. 

Second, patients always have a knowledge gap with physicians because of medical specialization [23]. If physicians have adequate intelligence and skills, then they can tactically communicate highly qualified diagnoses based on the patients’ education levels and understanding. Then, patients can easily understand what they need to do without exerting considerable effort. Moreover, using OHCs requires patients to possess various kinds of literacies, such as health and computer literacies, thereby affecting their use of health information obtained from OHCs [29]. However, most patients may lack the ability to meet the demands of using OHCs. Physicians’ competence in computer skills helps them to provide better services to their patients and teach their patients how to use OHCs. Consequently, patients with chronic diseases will perceive that they are capable of using OHCs for health advice.

**Hypothesis** **1** **(H1).** *Physicians’ competence has a positive effect on PU.*

**Hypothesis** **2** **(H2).** *Physicians’ competence has a positive effect on PEOU.*

### 2.4. Warmth

Warmth is an indicator used to measure the behavioral intention of others [13]. This indicator reflects the need for people to form and maintain social connections. Warmth is characterized by friendliness, helpfulness, trustworthiness, generosity, and communication [14]. In the medical industry, patients raised in the paternalistic phase of medicine have been conditioned to think of physicians as authority figures. Accordingly, warmth barely contributed to their preferences in the past. Evidently, patients dislike an overtly hostile physician. However, warmth is less often first prioritized for these patients. However, medical consultation is turning into a service industry, and the service attitude is increasingly becoming important [30,31]. Chronic patients are not being as serious as those of emergency patients, thus, they may have little urgent needs for the use of OHCs. Thus, the study assumes that physicians’ warmth has a positive effect on their PU and PEOU. 

As for PU, Parasuraman et al. [32] stated that given how services are intangible, heterogeneous, and indivisible, the intentions of service providers can be significantly different from how services are received by consumers. Thus, actual usefulness is different from perceived usefulness. In the context of health, medical resources cannot meet demands, and physicians are too busy to fully explain the reasons for treatment in detail [6]. Physicians struggle, in particular, to engage in face-to-face communication with chronic patients on the Internet. Sense making, giving and sharing are difficult to achieve without meeting in person [33]. Thus, physicians’ attitudes are critical for delivering treatment online. Warmth is related to service quality, and service quality is indispensable to service providers seeking to gain a competitive advantage and offer usefulness to consumers [34]. Physicians proactively engage in communicating with patients, and increased warmth can help demonstrate the value of health information by conveying it as comprehensible and positing it in a way that is as acceptable as possible for patients [33]. Specifically, physicians with warm characteristics may likely transfer a wealth of medical information to inform patients about the effectiveness of modern medical care in treating chronic health problems. Accordingly, physicians’ warmth possibly increases chronic patients’ perception that OHCs are useful for improving their current situation.

PEOU refers to the convenience and ease with which users can utilize technology online. On the one hand, support for IT will affect individual beliefs about the ease-of-use of IT [35]. As a social support, warmth can affect one’s computer self-efficacy and outcome expectations, which will, in turn, affect patients’ perceptions of OHC usage [36]. Yet, the strong professionalism of medical services often leads to an information asymmetry between physicians and patients [37], which is also one of the existing contradictions between physicians and patients. Patients may be confused about the health information provided by physicians and perceive that OHCs are difficult to use and not user friendly. If physicians are good natured and kindly explain details about a medical treatment, then patients are likely to understand their health condition and treatment, and also perceive that OHCs are easy to use [6]. As a result, physicians’ warmth can increase the possibility of patients’ PEOU. Furthermore, if physicians provide channels for timely and kind communication online, chronic patients will receive treatments without long waiting times. Then, patients will perceive the use of computers for medical consultation via OHCs to be easier when compared with visiting hospitals, given that the latter is time-consuming and costly.

**Hypothesis** **3** **(H3).** *Physicians’ warmth has a positive effect on PU.*

**Hypothesis** **4** **(H4).** *Physicians’ warmth has a positive effect on PEOU.*

### 2.5. PU, PEOU, and BIU

According to the TAM, individual BIU can be affected by PU and PEOU [38]. PE and PEOU can lead to a high reputation and lessen the information asymmetry between providers and consumers on the technology platform [39]. Meanwhile, PE and PEOU assist consumers in making reasonable decisions to reduce the level of perceived risk and uncertainty relating to OHCs. Thus, this study assumed that patients with chronic diseases will be engaged in using OHCs when they perceive that OHCs can improve their health condition, as well as being easy and user-friendly. 

On the one hand, health information contains data, such as a person’s health condition and medical history; usually, highly sensitive individuals conduct a risk-benefit analysis and determine the driving and inhibiting factors of technology acceptance [40]. OHCs are platforms used by health consumers and patients to discuss different diseases, therapeutic regimens, and personal experiences, especially for chronic diseases. Especially, in physician-centered OHCs, physicians’ engagement can guarantee the health information’s quality and the treatment effects. High engagement can support chronic patients to perceive the usefulness of OHCs in improving their health condition. Thus, patients will be more likely to select OHCs for better therapeutic effect, regardless of their level of information sensitivity.

On the other hand, previous studies on consumer adoption of online services have shown that PEOU is a key prerequisite for users to adopt new web technologies [41]. Specifically, the use of OHCs requires patients’ ability to use digital devices. Using OHCs requires users not only to have an understanding of health knowledge but also the ability to search for health information and make decisions about health behaviors. Whether the utilization of OHCs will be painless or effortless for chronic patients is related to the patients’ use intentions [42]. Hence, if patients perceive that OHCs are easy to use, then they may have the intent to use them.

In addition, TAM theory asserts that PEOU has a positive impact on PU. This study included this relationship in our model to create a complete network. Thus, we are specifically presenting the essential theoretical arguments presented in the existing literature. On that basis, the following hypotheses were formulated:

**Hypothesis** **5** **(H5).** *PU has a positive effect on chronic patients’ BIU OHCs.*

**Hypothesis** **6** **(H6).** *PEOU has a positive effect on chronic patients’ BIU OHCs.*

**Hypothesis** **7** **(H7).** *PEOU has a positive effect on PU.*

## 3. Materials and Methods

### 3.1. Instrument Development

A multiple-item measurement scale was used to measure the constructs (as shown in Appendix A Table A1). A seven-point Likert-type response format that ranged from “strongly disagree” to “strongly agree” or ranged from “not at all” to “extremely” was used to measure the items. The seven-point Likert scale provided additional choices, which in turn, increased the likelihood of meeting people’s objective reality. This scale revealed more descriptions of the subject and thus actually appealed to the participants’ rational competence [43]. To ensure reliability and validity, we used scales that have been used in published work. Chronic patients’ BIU all physician-centered OHCs was measured by a three-item scale obtained from the work of Egea et al. [44]. Perceived physicians’ competence and warmth were measured in accordance with the work of Fiske et al. [14] using a five-item scale. Perceived physicians’ competence and warmth were measured from patients’ experiences with their physicians in OHCs. However, each patient may communicate with multiple physicians. Thus, each patient was asked to make an overall judgement based on the average experience within three months. The scale items were modified in accordance with the work of Wu et al. [45] and Kim et al. [46] to measure PU, whereas the scale items from the work of Wu et al. [45] and Chang [47] were modified to measure PEOU.

### 3.2. Analysis Tool Selection

This study adopted partial least squares structural equation modeling to test the hypotheses. SmartPLS version 3.2.8 (SmartPLS GmbH, Bönningstedt, Germany) was used for the effective and unbiased analysis and assessment of potential variable interactions.

### 3.3. Data Collection and Respondent Profile

The formal investigation was anonymously conducted through a web-based questionnaire survey addressed to participants in December 2020. Our subjects were individuals who had received medical treatment in OHCs within the previous three months. Thus, all the respondents communicated with at least one particular physician in the OHCs. With the help of a medical association, we connected with individuals who had received medical treatment in the last three months in OHCs. We sent a web-based questionnaire to 1100 participants by email, mobile phone message, or social media. Depending on their experience of medical treatment with their physicians, the participants had impressions and judgements of their physicians’ characteristics, i.e., competence and warmth. Therefore, the subjects completed the questionnaires depending on their real experience and assessment of physicians and OHCs. Given that our subjects were Chinese, the study team conducted a translation process for cross-cultural adaptation. First, the scale items were translated into Chinese and then translated back into English by three professors independently to ensure the accuracy of translation. To improve the comprehensibility and readability, several physicians who had experience in OHCs and had different backgrounds in terms of age, gender, hospital level, and professional title were recruited to complete the questionnaire and provide modifications. Meanwhile, this study specified an additional qualification that respondents must have used physician-centered OHCs by defining screening questions to double-check their experience of the physician-centered OHCs. 

Before formal data collection, we performed a pilot survey of 33 patients to modify the questionnaire. A total of 897 responses were received, 710 of which were valid. (The web-based platform recorded the completion time of each response, and a response whose completion time was evidently lower than the average time was regarded as invalid. In addition, an incomplete response, i.e., that missing at least one answer, was invalid.) The response and validity rates were 81.5% (897/1100) and 79.2% (710/897), respectively. Gifts were given to participants as rewards (each valued at about one dollar). Table 1 shows the demographics of the sample. Most of the participants were aged 20–40 years (79.6%; 565/710), 65.4% (464/710) of the participants were female, and 82.3% (584/710) held at least a bachelor’s degree. The OHC user characteristics [48] were young, female, and highly educated. Thus, the scale met the requirements. 

## 4. Results

### 4.1. Descriptive Statistics 

This study added gender, age, living area, and education level as control variables. As shown in Table 2, the reliability of the scales was measured by calculating Cronbach’s alpha. The reliability was acceptable given that the value of each construct was greater than the cut-off value of 0.700. The convergent validity of scales was acceptable because all the composite reliability (CR) values were greater than the cut-off value of 0.700, and all the average variance extracted (AVE) values were greater than the cut-off value of 0.500. Table 3 shows the results of the Fornell–Larcker test of discriminant validity. The discriminant validity was acceptable because the square root of AVE (values in the diagonal) was always above the corresponding line and row values in the diagonal (the correlations between each of the two constructs) [49]. 

### 4.2. Hypothesis Testing 

Demographical statistics were calculated to identify any significant relationship between the demographic factors and variables of the research model. This study calculated the multivariate coefficient of determination (*R*^2^) of variables with and without control variables. As shown in Table 4, depending on the results of *R*^2^, Cohen *f*^2^ [1,50] was used to assess the effects of control variables (i.e., insignificant: <0.020; small: ≥0.020 and <0.150; medium: ≥0.150 and <0.300; large: ≥0.350). The effect sizes of control variables were all insignificant. Thus, control variables all had limited effects on the constructs.

**Figure 2:**
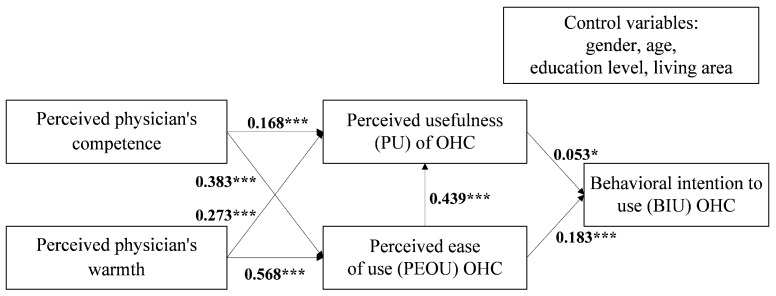
Research model with path coefficients. *** *p* < 0.001; * *p* < 0.05.

Table 5 and Figure 2 present the magnitude and significance of the path coefficients, respectively. All seven hypotheses were supported, and all relationships were positive. However, the effect of PU on BIU was weak.

In addition, this study analyzed the mediating effects by using the bootstrapping method (*n* = 5000, 95% confidence interval (CI)). As shown in Table 6, the indirect effects were significant, and the CIs excluded zero. Thus, the mediating effects were significant.

## 5. Discussion

### 5.1. Principal Findings

This study is one of the first to explore the effect of physicians’ traits on chronic patients’ intention to use OHCs from the perspectives of competence and warmth, and it enriches the research on physician-centered OHCs. First, this study constructed a research model for the physicians’ traits and chronic patients’ BIU, as mediated by PU and PEOU. The competence and warmth of physicians had a positive and significant effect on patients’ BIU through the mediating factors of PU and PEOU. Existing studies have investigated and discovered various determinants of patients’ intention to use online health services from their perspective, such as perceived behavior control, perceived severity of disease [51], health literacy [52], etc. The findings proved that physicians also impact patients’ intention to use OHCs. Meanwhile, the findings corresponded with those of previous studies, showing that individual social cognition of others can be classified into competence and warmth [14]. Therefore, this study enriches the theoretical research on OHCs and physicians’ traits. 

Second, competence and warmth positively impacted PU and PEOU. However, warmth had a slightly stronger impact than competence. This finding shows that chronic patients place a higher value on physicians’ warmth than their competence. Notably, warmth as a priority also depends, to a certain extent, on the question being asked. A radiologist who lacks care for a patient will be likely viewed as a “black box” where technical answers alone come back; here, technical skill is the primary concern and dealing with a chronic patient evidently needs substantial amount of warmth to gain their trust. A previous study [53] identified the psychological characteristics of chronic patients: anxiety, depression, and pessimism. Chronic patients are always plagued by illness. Thus, they need extra care and concern from their physicians. Thus, physicians should not only pay attention to their professional abilities but also use influencing tactics to show their warmth. 

Third, BIU is positively affected by PU and PEOU, and PEOU has a positive effect on PU. However, the impact of PU on BIU is smaller (0.053, *p* = 0.046) than that of PEOU (0.183, *p* < 0.001). A suppression effect by other factors possibly exists. Chronic patients with a low level of eHealth literacy are unlikely to use OHCs whether they perceive OHCs as useful. Further studies can explore the moderate effect of eHealth literacy. This finding indicates that OHC designers should pay more attention to enhancing their user friendliness for patients with chronic diseases [54]. Specifically, technology designers are advised to promote human–computer interaction by clarifying the logic involved and making the interface and website flow concisely. Then, users will perceive OHCs as easy to use. Moreover, given the unexpected results regarding the weak relationship between PU and BIU, gaps may exist between actual and perceived outcomes [55]. OHC designers are required to understand the users’ actual attitudes toward usefulness.

### 5.2. Implications for Practice

This study has two implications for practice. First, the study showed that physicians have impacts on patients’ intention to use OHCs. The findings highlight the promise of physician-driven OHCs in facilitating collaborative care between patients and physicians. OHC policymakers should realize physicians’ effects on patients’ usage intention. The ongoing integration of physicians’ medical expertise and affinity to physician–patient OHCs drives the willingness of patients to continue using OHCs. Physicians can engage patients in health-promoting behaviors, including seeking health information and emotional support, expressing emotions, and sharing their consultation experiences, through the use of OHCs. Specifically, OHCs can strengthen the management of physicians’ quality from the perspective of competence and warmth. Our model can also be adopted in other domains to address the problems of users’ intention to use OHCs from the perspective of the competence and warmth of service staff. 

OHC managers and physicians should pay attention to the higher priority given by chronic patients to physicians’ warmth than competence. Traditional care often views patients as passive recipients of medical services and ignores the emotional support needed by them. In non-acute cases, such as mental health, recovery from a disease requires a long period of self-management, in which care-making decisions are tailored to the patient’s circumstances. In the patient–physician partnership for chronic diseases, physicians and patients are both specialists, i.e., physicians specialize in medicine and patients specialize in their life. Thus, physicians should collaborate with patients and give them emotional support. Physicians should explain the pathogenesis of chronic patients and their therapeutic regimen while avoiding the use of terminologies. Meanwhile, OHCs should enact measures to guarantee the timely responses of physicians, such as limiting the physicians’ maximum response time and increasing the numbers of physicians.

### 5.3. Limitations and Prospects

The limitations of the study must be considered. First, this study only focused on two dimensions of physicians’ traits: competence and warmth. Other dimensions may also be investigated. Physicians’ competence can be discussed in detail, focusing on physicians’ communication skills, health literacy, computer literacy, etc. Second, this study collected data by a cross-sectional survey. Thus, it lacked the process of capturing the changes in participants’ attitudes toward all variables. Third, although our sample matched the characteristics of OHC users, the effect of OHCs in China may differ from that in other countries. Thus, the findings should be further investigated in other countries to generalize our findings to the populations of all personal OHC users in the world. Fourth, our sample size was 710, which is larger than that in most similar studies. However, the collected sample may be insufficient considering the Chinese population with regard to all the available OHCs in China. Further research should collect more data to verify the results. Finally, we used TAM theory to analyze the research model. The unified theory acceptance and use of technology (UTAUT 2) [56] is a more sophisticated and comprehensive model for the investigation of individual attitudes toward technology. Further studies can use UTAUT2 to confirm the antecedents of individuals’ intentions to use OHCs.

## 6. Conclusions

This study indicates that physicians’ competence and warmth have positive effects on chronic patients’ BIU OHCs through the mediation of PU and PEOU based on the TAM theory. This research is one of the first attempts to explore the cognitive activities of chronic patients from the perspective of physicians’ competence and warmth. The findings suggest the following: (1) Further studies can focus on how to improve patients’ usage attitudes and behaviors from the perspective of physicians. (2) Improving physicians’ professional capabilities for treatment is insufficient; warmth and kindness should be paid more attention, with the intent of benefiting patients who bestow it a higher priority, especially patients with chronic disease. (3) The ease of use of OHCs is more valuable for chronic patients when compared with their usefulness; designers of physician-centered OHCs should, accordingly, study how to design a user-friendly interface rather than improve the usefulness of OHCs.

## Figures and Tables

**Table 1:** Sample demographics (*n* = 710).

Demographic Characteristics	Participants, *n* (%)
Age (years)	
<20	26 (3.7)
20–29	342 (48.2)
30–39	223 (31.4)
40–49	57 (8.0)
50–59	47 (6.6)
60 and above	15 (2.1)
Gender	
Male	246 (34.6)
Female	464 (65.4)
Resident status	
Urban	509 (71.7)
Rural	201 (28.3)
Education	
Junior middle school	15 (2.1)
High school	33 (4.6)
Junior college	78 (11.0
Bachelor’s degree	368 (51.8)
Master’s degree	198 (27.9)
Physician’s degree	18 (2.5)

**Table 2:** Cronbach’s alpha, CR, AVE, and square root of AVE.

Construct	Cronbach’s Alpha	CR	AVE	Square Root of AVE
Competence	0.905	0.929	0.775	0.880
Warmth	0.966	0.973	0.880	0.938
PU	0.722	0.840	0.728	0.853
PEOU	0.912	0.945	0.851	0.922
BIU	0.932	0.956	0.880	0.938

**Table 3:** Fornell–Larcker test of discriminant validity.

Construct	Competence	Warmth	PU	PEOU	BIU
Competence	0.880	0.853	0.781	0.867	0.877
Warmth		0.938	0.809	0.894	0.911
PU			0.853	0.829	0.825
PEOU				0.922	0.916
BIU					0.938

**Table 4:** Multivariate coefficient of determination (*R*^2^) results.

Variables	R Square		Control Variables Effects		
	With Control Variables	Without Control Variables	Δ*R*^2^ ^a^	*f* ^2^ ^b^	Effect
BIU	0.918	0.917	0.001	0.001	Insignificant
PEOU	0.840	0.840	<0.001	<0.001	Insignificant
PU	0.716	0.716	<0.001	<0.001	Insignificant

^a^ Δ*R*^2^: *R*^2^ with control variables—*R*^2^ without control variables; ^b^ *f*^2^: Cohen *f*^2^.

**Table 5:** Hypothesis testing.

Hypothesis	Path Coefficient	*t* Test	*p* Value
H1: Competence → PU	0.168	4.218	<0.001
H2: Competence → PEOU	0.383	9.590	<0.001
H3: Warmth → PU	0.273	5.630	<0.001
H4: Warmth → PEOU	0.568	14.482	<0.001
H5: PU → BIU	0.053	1.999	0.046
H6: PEOU → BIU	0.183	5.003	<0.001
H7: PEOU → PU	0.439	8.277	<0.001

**Table 6:** Path coefficients by bootstrapping.

Effect	Path Coefficients	*p* Value	CI
Direct effect			
Competence → PU	0.168	<0.001	0.092–0.251
Competence → PEOU	0.383	<0.001	0.305–0.461
Warmth → PU	0.273	<0.001	0.177–0.367
Warmth → PEOU	0.568	<0.001	0.490–0.643
PU → BIU	0.053	0.046	0.003–0.106
PEOU → BIU	0.183	<0.001	0.110–0.254
PEOU → PU	0.439	<0.001	0.332–0.539
Indirect effect			
Competence → BIU	0.088	<0.001	0.053–0.128
Warmth → BIU	0.131	<0.001	0.082–0.183
Total effect			
Competence → BIU	0.088	<0.001	0.053–0.128
Warmth → BIU	0.131	<0.001	0.082–0.183

## Abbreviations

PU: Perceived usefulness; PEOU: Perceived ease of use; BIU: Behavioral intention to use; OHC: Online health community.

## Appendix A

1. Have you received any medical treatment from a physician-centered online health community (OHC) in the last three months?

—Yes —No

**Table A1:** Measurement instruments.

Construct	Scale/Scoring	Items
Behavioral intention to use	Seven-point Likert Strongly disagree to strongly agree	BIU1: Assuming that I am given the chance to access telemedicine, I intend to use physician-centered OHC services.
		BIU2: Whenever I need remote medical care from professionals, I would gladly use physician-centered OHCs.
		BIU3: I intend to inform my relatives and friends about physician-centered OHCs.
Perceived usefulness	Seven-point Likert Strongly disagree to strongly agree	PU1: Using physician-centered OHCs would improve the quality of my healthcare.
		PU2: Using physician-centered OHCs would improve my access to healthcare services.
		PU3: Using physician-centered OHCs would be useful in my daily routine.
Perceived ease of use	Seven-point Likert Strongly disagree to strongly agree	PEOU1: I find learning to use physician-centered OHCs to be easy.
		PEOU2: I find it easy to interact with doctors using physician-centered OHCs.
		PEOU3: Interacting with physician-centered OHCs is clear and understandable for me.
Competence	Seven-point Likert Not at all to extremely	According to your online treatment experience with physician(s) in physician-centered OHCs:
		C1: How competent are physician(s)?
		C2: How confident are physician(s)?
		C3: How independent are physician(s)?
		C4: How competitive are physician(s)?
		C5: How intelligent are physician(s)?
Warmth	Seven-point Likert Not at all to extremely	According to your online treatment experience with physician(s) in physician-centered OHCs:
		C1: How tolerant are physician(s)?
		C2: How warm are physician(s)?
		C3: How good natured are physician(s)?
		C4: How sincere are physician(s)?
